# Implementation of non-insulin-dependent diabetes self-management education (DSME) in LMICs: a systematic review of cost, adoption, acceptability, and fidelity in resource-constrained settings

**DOI:** 10.3389/frhs.2023.1155911

**Published:** 2023-06-13

**Authors:** Reilly Fitzpatrick, Shubhra Pant, Jamie Li, Rebecca Ritterman, Deborah Adenikinju, Chukwuemeka Iloegbu, John Pateña, Dorice Vieira, Joyce Gyamfi, Emmanuel Peprah

**Affiliations:** ^1^ NYU School of Global Public Health, New York, NY, United States; ^2^ Global Health Program, Department of Social and Behavioral Sciences, ISEE Lab, NYU School of Global Public Health, New York, NY, United States; ^3^ NYU Health Sciences Library, NYU Grossman School of Medicine, New York, NY, United States

**Keywords:** LMIC, T2D, DSME, implementation outcomes, cost, adoption, acceptability, fidelity

## Abstract

**Background:**

Type II diabetes (T2D), is a serious health issue accounting for 10.7% of mortality globally. 80% of cases worldwide are found in low- and middle-income countries (LMIC), with rapidly increasing prevalence. Diabetes-self management education (DSME) is a cost-effective program that provides at-risk individuals with the knowledge and skills they need to adopt lifestyle changes that will improve their health and well-being. This systematic review examined the application of DSME in LMICs and identified the corresponding implementation results (cost, fidelity, acceptance, and adoption) associated with successful implementation in low-resource settings.

**Methods and analysis:**

The available research on T2D and the use of DSME in LMIC were systematically searched for using six electronic databases (PubMed, Embase, Cochrane, Web of Science, Google Scholar, PAIS, and EBSCO Discovery) between the months of October and November of 2022. The articles that met the search criteria were subsequently imported into EndNote and Covidence for analysis. The Cochrane RoB methodology for randomized trials was used to evaluate the risk of bias (RoB) in the included studies. A narrative synthesis was used to summarize the results.

**Results:**

A total of 773 studies were imported for screening, after 203 duplicates were removed, 570 remained. Abstract and title screenings resulted in the exclusion of 487 articles, leaving 83 for full-text review. Following a full-text review, 76 articles were excluded and seven were found to be relevant to our search. The most common reasons for exclusion were study design (*n* = 23), lack of results (*n* = 14), and wrong patient population (*n* = 12).

**Conclusion:**

Our systemic review found that DSME can be an acceptable and cost-effective solution in LMIC. While we intended to analyze cost, adoption, acceptability, and fidelity, our investigation revealed a gap in the literature on those areas, with most studies focusing on acceptability and cost and no studies identifying fidelity or adoption. To further evaluate the efficacy of DSME and enhance health outcomes for T2D in LMICs, more research is needed on its application.

**Systematic Review Registration:**

osf.io/7482t.

## Introduction

Non-insulin-dependent diabetes, commonly known as type II diabetes (T2D), is a growing health challenge globally (1). Nearly 540 million adults ages 20–79 live with diabetes, accounting for 10.7% of all-cause mortality (2). Nearly 80% of those living with diabetes reside in low and middle-income countries (LMICs) and rates continue to rise rapidly compared to high-income countries (HICs) (3). By 2035, diabetes prevalence is projected to increase by 73% in LMICs, compared to 28% in HICs (4, 5). The rate of increase in diabetes is inversely related to the countries' income status, as rapid urbanization, and economic development in LMICs have initiated the adoption of dietary habits and lifestyle choices associated with disease development (1, 6, 7).

T2D is the most common form of diabetes and represents 90% of cases globally (8). The chronic condition is characterized by an irregular physiological response to insulin (9). Lifestyle risk factors contributing to the development of TD2 include obesity/overweight, inactivity, diet, and hypertension (10). Uncontrolled type two diabetes can cause hyperglycemia, or high blood sugar, leading to disabling micro and macrovascular complications (11). According to data from Institute for Health Metric and Evaluation, T2D represents two point 5% of total disability-adjusted life years (DALY) in LMICs, with an annual charge of two point one-eight percent (12). However, individuals in LMICs die from diabetes-related complications that are often registered as other conditions, thus underestimating the true impact of diabetes on the population (13).

Education focusing on lifestyle interventions is a critical component of diabetes treatment for at-risk or diabetic populations (13). Diabetes-self management education (DSME) is an evidence-based intervention that empowers at-risk individuals with the knowledge and skills to make lifestyle changes that promote health and well-being (14). Self-management includes behaviors such as healthy eating, physical activity, medication usage, and detection and treatment of complications related to the disease (15).

DSME is a cost-effective intervention that can lead to a 30%–60% relative reduction in diabetes incidence and 47% and 41% long-term reductions in retinopathy and cardiovascular mortality, respectively (16). Such programs have been tested successfully in LMICs. Patients enrolled in DSME programs in the World Health Organization (WHO) African Region have shown statistically significant improvements in blood glucose (HbA1c levels), blood pressure, and diabetes-related knowledge (14). Moreover, in a scoping review of self-management in Sub-Saharan Africa (SSA), Stephani et al. (17) identified six studies of DSME programs that showed significant improvements in diet and activity habits, medication adherence, and risk reduction behavior.

Despite the reported benefits of DSME in LMICs, a vast majority of the research conducted on diabetes education programs has been in HICs. In a recent systematic review, Lamptey et al. (18) found a significant dearth of evidence showing the effectiveness of structured diabetes education in LMICs. Furthermore, the lack of culturally appropriate prevention programs threatens the acceptability of the intervention (19). For example, Stephanie et al. (17) found that the “western” model of DSME failed to represent the self-care activities in SSA. The review showed that one-third of all patients in SSA sought alternative medicine in addition to their biomedical therapy, compared to just eight percent in non-SSA countries. The disproportionate underfunding of diabetes prevention in LMICs is also a significant contributor to the underutilization of DSME programs in regions where the burden is significant and populations could benefit (20). Additionally, a severe shortage of human resources and trained healthcare providers in LMICs creates a diabetes management care gap (17). Implementation of DSME may mitigate some of these issues in resource-constrained settings, such as LMICs. Our rationale for undertaking a systematic review was to explore the implementation of diabetes self-management education in LMICs and determine the cost, fidelity, acceptability, and adoption to successfully implement evidence-based education programs in low-resource settings.

## Methods

### Search strategy

We conducted a comprehensive search of six electronic databases (PubMed, Embase, Cochrane, Web of Science, Google Scholar, PAIS, and EBSCO Discovery). A research librarian supported the development of a search strategy. The search strategy included Medical Subject Heading (MeSH) terms as well as other key terms for the main subjects “diabetes self-management education”, “non-insulin-dependent diabetes” and the implementation outcomes, and “low and middle-income countries as defined by the World Bank”. The complete search strategy for all six databases can be found in the Appendix. The search was conducted from October through November 2022, and the resulting articles were imported into EndNote and then into Covidence.

### Inclusion and exclusion criteria

We utilized the Population, Intervention, Control, Outcome (i.e., PICO) format to guide our search strategy. Studies were included if they met the following inclusion criteria: (1) were published RCTs implemented in LMICs, (2) reported on non-insulin-dependent diabetes management in LMICs, (3) examined only non-complicated cases of diabetes, (4) measured implementation outcomes including cost, fidelity, acceptability, and/or adoption (5) were published in English. Non-English studies were excluded due to time and human resource constraints. No restrictions were placed on publication year, and non-randomized studies, protocols, commentaries, and other reviews were excluded in order to review the highest-quality data.

### Data extraction

All citations were downloaded to EndNote and then Covidence for the title and abstract screening. All four group members independently screened 83 studies/articles to determine if they met the inclusion criteria. We obtained the full-text article and reviewed them independently. Any conflict regarding the exclusion of relevant information was extracted from the full-text article. Specifically, the following study characteristics were retrieved and coded: intervention type, duration, intervention setting, country, sample size, and implementation outcomes (cost, fidelity, acceptability, adoption). Proctor et al.'s (21) definition of implementation outcomes was used to identify relevant information from the eligible articles. Identification of implementation outcomes required in-text review, as many of the recorded outcomes were secondary measures.

## Results

### Literature search

**Figure 1:**
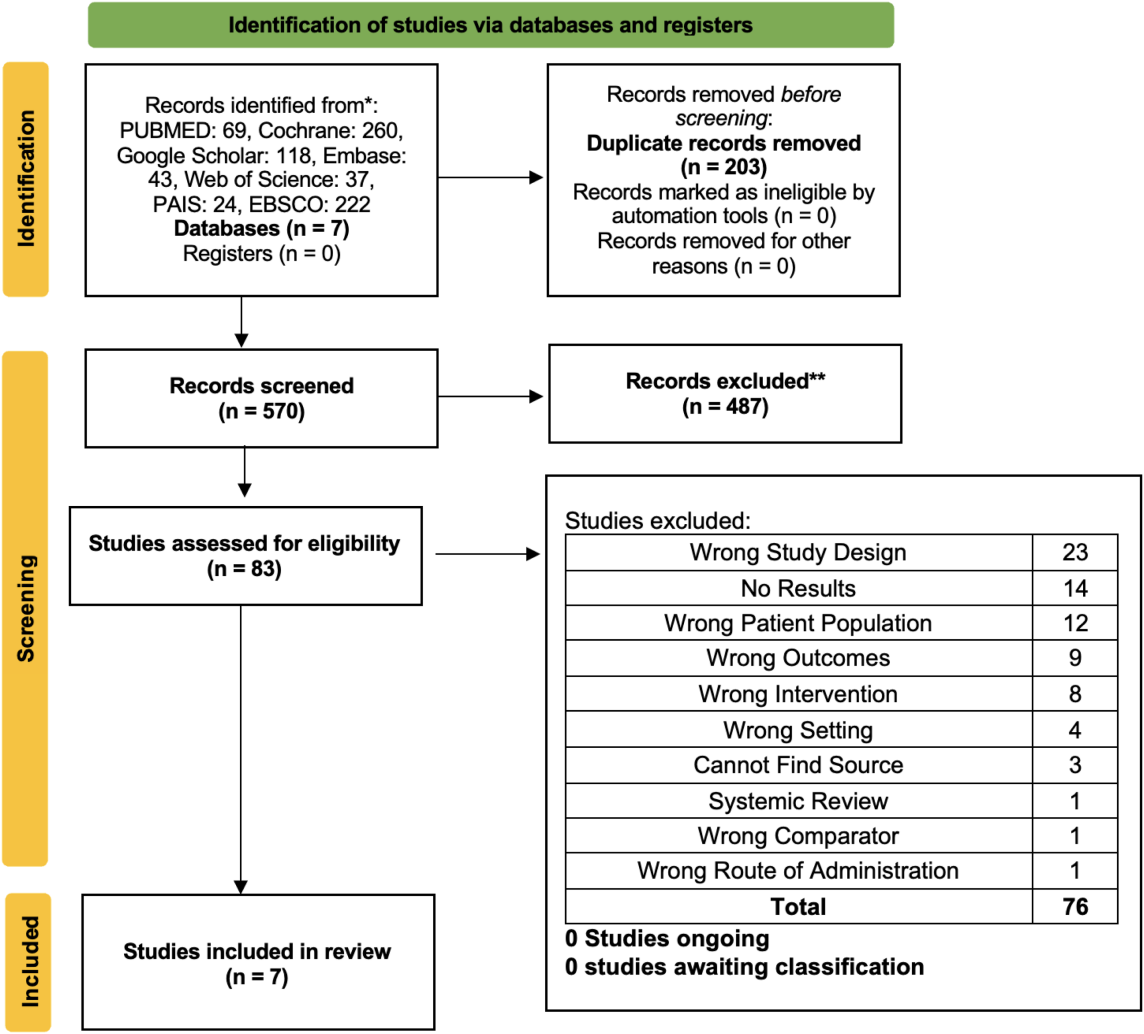
PRISMA flowchart.

A total of 773 studies were imported for screening. After 203 duplicates were removed, 570 studies remained. The abstracts and titles screening resulted in the exclusion of 487 articles, leaving 83 for full-text screening. Following a full-text review, 76 articles were excluded and seven were found to be relevant to our review. The most common reasons for exclusion were study design (*n* = 23), lack of results (*n* = 14), and wrong outcomes (*n* = 12). The process of study identification and selection and the reasons for exclusion are depicted in Figure 1.

### Characteristics of identified studies

The studies selected were published between 2013 and 2021. The mean duration of the studies was approximately four months (min two—max six months). Over two-thirds of the studies identified were in Africa, *n* = 5 (71%), and two out of the seven studies were in Asia. Nearly all of the studies were conducted in middle-income countries, as classified by the World Bank; *n* = 6, with only one study conducted in a low-income country. The studies were composed of both men and women over the age of 18 and under 65. All studies were conducted through urban health centers. Out of the seven studies, several different vehicles were used to implement the DSME. Three out of the seven studies used mobile messaging (SMS) to educate individuals in the intervention group. Two out of seven studies examined the effect of education provided by clinical pharmacists, and the final two studies evaluated the impact of group DSME sessions.

### Implementation outcomes

Acceptability was the most frequently discussed implementation outcome (*n* = six), followed by cost (*n* = one). None of the studies identified discussed fidelity or adoption. All studies identified assessed implementation outcomes at the patient level (*n* = seven). Overall, satisfaction with the various DSME interventions was high. In the three studies using SMS education, average patient satisfaction was 93.4% among participants receiving the intervention. Moreover, in Abaza et al. (22) 100% of patients indicated satisfaction with daily educational text messages and said they would recommend the program to others. Two studies assessed the acceptability of the intervention by comparing diabetes satisfaction ratings between the control and treatment groups. In Simon et al. (23), the Diabetes Treatment Satisfaction Questionnaire (DTSQ) was used to assess the acceptability of DSME provided by a clinical pharmacist. Following one educational session with a pharmacist, the patient's DTSQ score significantly improved in the intervention group compared to the control. In Shakibazadeh et al. (24), patients who attended ten Persian Diabetes Self-Management Education (PDSME) workshops were significantly more satisfied with diabetes care compared with patients receiving usual care at two and eight weeks (*p* < 0.001). Hailu et al. (25) used patient retention and attendance as a proxy for the program's acceptability. Less than 40% of participants completed the program, and over a third reported difficulty attending the sessions due to transportation issues and financial constraints. Adibe et al. (26) was the only study that discussed cost. In the study, researchers explored the cost-effectiveness of a pharmacist-led diabetes education program compared to standard diabetes care. The study relied on WHO thresholds for cost-effectiveness that vary by region. Findings from this study indicated that the pharmacist-led intervention resulted in an incremental gain in quality-adjusted life years and cost compared with usual care.

### Risk of bias assessment

The seven RCTs were evaluated using the Cochrane Risk of Bias tool. Cochrane's risk of bias tool assesses studies for random sequence generation (selection bias), allocation concealment (selection bias), blinding of participants and personnel (performance bias), blinding of outcome assessment (detection bias), incomplete outcome data (attrition bias), and selective reporting (reporting bias). A study flagged as low-risk indicated that the item was well described and accounted for in the study; high risk of bias indicated an insufficient description of the item in the study, and unclear risk of bias indicated that there was no information provided in the article to enable determination of the specific item of bias. Combining random sequence generation and allocation concealment results in a selection bias. Random sequence generation caused a low risk of selection bias in 71% of the studies, and allocation concealment caused a low risk of selection bias in around 85% of the studies. Due to the participant and staff blinding, 57% of studies had a low risk of performance bias; slightly more than 47% of studies had a low risk of detection bias, and nearly 47% of studies had a low risk of attrition bias due to insufficient outcome data. A low risk of reporting bias was present in over 17% of the studies because of selection bias (Table 1).

**Table 1:** Risk of bias Assessment.

Author/year	Random sequence generation	Allocation concealment	Appraisal [blinding of participants]	Blinding of personnel	Blinding of outcome assessors	Incomplete outcome data [attrition]	Selective reporting
Abaza and Marschollek (22)	Low	Low	Low	Unsure	Low	Low	High
Adibe et al. (26)	Low	Low	High	Low	High	Low	High
Asante et al. (27)	Low	Low	Low	High	Unsure	High	High
Hailu et al. (25)	High	Low	High	High	High	Low	High
Owolabi et al. (28)	High	High	High	High	Low	Low	High
Shakibazadeh et al. (24)	Low	Low	Low	Low	High	High	Low
Simon et al. (23)	Low	Low	Low	Low	Low	High	High

## Discussion

This is the first systematic review to our knowledge to evaluate the acceptability, cost, fidelity, and adoption of diabetes self-management education (DSME) in LMICs. Our comprehensive review revealed a serious shortage in implementation research in this area, as indicated in the seven articles included. While we sought to evaluate cost, adoption, acceptability, and fidelity, the existing literature concentrates on acceptability and cost, and no studies measuring fidelity or adoption were identified. However, given that there is just one publication detailing cost (Refer Table 2), DSME's reliability as a cost-effective treatment method needs to be further investigated.

**Table 2:** Study characteristics and Implementation outcomes.

Study	Study design	Study duration (months)	Country	Sample size	Description of intervention	IS outcome	IS outcome synthesis
Abaza and Marschollek (22)	RCT	3	Egypt	90	Daily educational text messages for a total of 84 messages per patient.	Acceptability	100% of patients indicated general satisfaction with the program said they would stay enrolled should the program continue, & would also recommend it to others.
Adibe et al. (26)	RCT	3	Nigeria	220	Four DSME sessions with pharmacists for 90–120 min.	Cost	Very cost-effective among patients at an NGN 88,600 ($572) per QALY gained threshold
Asante et al. (27)	RCT	3	Ghana	60	12 weeks of mobile phone educational calls by a nurse with a mean duration of 12 min each.	Acceptability	Participants who received the intervention rated their satisfaction as 89.3% (8.93/10) on average.
Hailu et al. (25)	RCT	6	Ethiopia	120	Six educational sessions, each lasting for 1.5 h on average; colorful, well-illustrated educational handbooks and fliers adapted to the local context; and extensive and interactive discussions with peers and take-home activities.	Acceptability	Only 39% of participants completed all 6 DSME sessions. One-third of the intervention group found it difficult to attend the DSME sessions consistently. Possible factors included age, food insecurity, and lack of reliable medications at the hospital.
Owolabi et al. (28)	RCT	6	South Africa	216	Daily educational SMSs. The intervention group received 184 SMS in total.	Acceptability	98/108 (90.74%) participants were satisfied with the intervention and felt it was helpful. Of those who participated in the intervention, 91% completed the follow-up after 6 months.
Shakibazadeh et al. (24)	RCT	2	Iran	280	Persian-DSME included eight 2.5 h educational workshops offered over a 4-week period followed by two “booster” sessions, each 2 weeks apart.	Acceptability	The PDSME patients were more satisfied with diabetes care compared with patients receiving usual care at 2 and 8 weeks (*p* < 0.001), and their satisfaction improved at 8 weeks compared with 2 weeks (*p* < 0.001).
Simon et al. (23)	RCT	6	India	97	One diabetes educational session with clinical pharmacist	Acceptability	The mean DTSQ (Diabetes Treatment Satisfaction Questionnaire) score significantly improved in the intervention group compared to the control.

The use of mobile technology in healthcare has had a positive impact on population health globally (29). The spread of mobile technology in LMICs supports innovative solutions to improve health in resource-constrained countries. Our review indicates that mobile messaging was an acceptable intervention for communicating DSME in LMICs. In the three studies identified, patient satisfaction rates were high, falling between 90%–100%. Moreover, the studies took place in both middle-income (South Africa) and lower-middle-income (Egypt and Ghana) countries, suggesting that results may be generalizable to various LMICs. To enhance patient satisfaction, Jain et al.’s (30) “qualitative review of patients” perspectives towards technology-assisted diabetes self-management education suggests that technologies should be easy to access, use, and apply and have reliable information.

The importance of culturally-appropriate programming in patient satisfaction was a key theme that emerged from the review. Shakibazadeh et al. (24) made modifications to the DSME program based on certain patients barriers that emerged during qualitative interviews The interviews revealed the importance of family support in patient satisfaction and self-efficacy Shakibazadeh (31), and thus modifications were made to include family members in sessions. In comparison, Hailu et al. (25) struggled with program adherence due to potential sociocultural factors the intervention did not account for. The authors suggest that low literacy rates and the cost of transportation impacted engagement with the program.

In contrast to many HICs, pharmacists are an underutilized resource in LMICs and may serve a vital role in under-resourced health systems (32). The review suggests that pharmacy-led DSME may be a cost-effective approach to TD2 management in LMICs such as Nigeria (26). This is in line with recent research by (33) that demonstrates pharmacist-led therapies save long-term expenses by enhancing glycemic control and lowering complications connected to diabetes (2016). C ost-effectiveness of pharmacy-led diabetes management services found that these interventions increased QALYs at reduced costs and saved $7 to $65,000 ($8 to $85,000 in 2014 US dollars) per person per year. However, it is crucial to remember that just two of the 25 research findings were carried out in LMICs.

Pharmacist-led DSME interventions may also enhance patient satisfaction (acceptability) with diabetes management (23). Following one diabetes educational session with a clinical pharmacist, Simon et al. (23) showed a significant improvement in participants' mean DTSQ (Diabetes Treatment Satisfaction Questionnaire). While this is the only RCT to our knowledge that measures patient satisfaction with pharmacist-led DSME in LMICs, quasi-experimental studies in LMICs have shown similar benefits. Abubkar et al. (34) showed a significant improvement in satisfaction (*p* = 0.04) as measured by a modified Diabetes Disease State Management Questionnaire, in patients enrolled in a six-month pharmacist-led intervention program in Pakistan.

### Limitations

There were several limitations that may have impacted the results of this review. The articles included conducted interventions over relatively short periods of time (between two and six months) Additionally, the studies' sample sizes were modest, which limited the generalizability necessary for RCTs to prove clinical efficacy/effectiveness (22, 35). While the studies included were based on LMICs, they were conducted through hospitals or research institutions in urban settings. As a result, we cannot conclude that this intervention would be implemented successfully in less-resourced settings such as rural health clinics within LMICs. This systematic review excluded non-randomized studies. While RCTs are the gold standard, integrating both quantitative and qualitative studies helps contextualize the research and provides a better understanding of the appropriateness of the intervention in a given setting (36). This review also limited our search to English publications. As our search was limited to LMICs, where English may not be the primary language, it is possible that some evidence may have been missed.

## Conclusion

Our systematic review revealed that DSME can be a cost-effective and acceptable intervention in LMICs. While our study aimed to also examine fidelity and adoption, our review failed to identify randomized controlled trials measuring these outcomes in LMICs. There is a need for more research on the implementation of DSME in LMICs in order to assess these as well as the additional outcomes defined by Procter et al. More research in this area will strengthen the effectiveness of DSME and improve health outcomes for T2D in LMICs.

## Data Availability

The datasets presented in this study can be found in online repositories. The names of the repository/repositories and accession number(s) can be found in the article.

## Author's note

At the time of writing, RF, SP, JL, and RR were MPH Candidates at the NYU School of Global Public Health, and have since graduated.

## Appendix sample search strategy

### LMICs

(“LMICs” [tw] OR “LMIC” [tw] OR “developing countries” [tw] OR “developing country” [tw] OR “medically underserved area” [tw] OR “medically underserved areas” [tw] OR “low income countries” [tw] OR “low income country” [tw] OR “middle income countries” [tw] OR “middle income country” [tw] OR “Global South” [tw] OR “resource poor” [tw] OR “low resource” [tw] OR “third world country” [tw] OR “third world countries” [tw] OR Africa OR Central Asia OR Western Asia OR Southeastern Asia OR Indian Ocean Islands OR Central America OR South America OR Eastern Europe OR Transcaucasia OR China OR Korea OR Mongolia OR Mexico OR Caribbean Region OR Pacific Islands OR Africa OR Central Asia OR Western Asia OR Southeastern Asia OR Indian Ocean Islands OR Central America OR South America OR Eastern Europe OR Transcaucasia OR Caribbean OR Pacific Islands OR Afghan OR Afghani OR Afghanistan OR Bangladesh OR Bangladeshi OR Benin OR Beninese OR Burkina Faso OR Burkinabe OR Burundi OR Burundian OR Cambodia OR Cambodian OR Central African Republic OR Central African OR Chad OR Chadian OR Comoros OR Comoran OR Congo OR Congolese OR Eritrea OR Eritrean OR Ethiopia OR Ethiopian OR Gambia OR Gambian OR Guinea OR Guinean OR Haiti OR Haitian OR Kenya OR Kenyan OR Korea OR Korean OR Kyrgyz OR Kyrgyzstan OR Liberia OR Liberian OR Madagascar OR Malagasy OR Malawi OR Malawian OR Mali OR Malian OR Mozambique OR Mozambican OR Myanmar OR Myanmarese OR Burmese OR Nepal OR Nepalese OR Niger OR Nigerian OR Rwanda OR Rwandan OR Sierra Leone OR Sierra Leonean OR Somalia OR Somalian OR Tajikistan OR Tajik OR Tadzhik OR Tanzania OR Tanzanian OR Togo OR Togolese OR Uganda OR Ugandan OR Zimbabwe OR Zimbabwean OR Angola OR Angolan OR Armenia OR Armenian OR Belize OR Belizean OR Bhutan OR Bhutanese OR Bolivia OR Bolivian OR Cameroon OR Cameroonian OR Cape Verde OR Cape Verdian OR Cape Verdean OR Cote d'Ivoire OR Ivory Coast Ivorian OR Djibouti OR Egypt OR Egyptian OR El Salvador OR Salvadorian OR Salvadorans OR Fiji OR Fijian OR Georgia OR Georgian OR Ghana OR Ghanaian OR Guatemala OR Guatemalan OR Guyana OR Guyanese OR Honduras OR Honduran OR Indonesia OR Indonesian OR India OR Indian OR Iraq OR Iraqi OR Kiribati OR Kosovo OR Kosovar OR Laos OR Lao OR Laotian OR Lesotho OR Marshall Islands OR Marshallese OR Mauritania OR Mauritanian OR Micronesia OR Micronesian OR Moldova OR Moldovan OR Mongolia OR Mongolian OR Morocco OR Moroccan OR Nicaragua OR Nicaraguan OR Nigeria OR Pakistan OR Pakistani OR Papua New Guinea OR Papua New Guinean OR Paraguay OR Paraguayan OR Philippines OR Filipino OR Samoa OR Samoan OR “Sao Tome and Principe” OR “Sao Tome and Principe” OR Senegal OR Senegalese OR Solomon Islands OR Solomon Islander OR Sri Lanka OR Sri Lankan OR Sudan OR Sudanese OR Eswatini OR Swazi OR Swaziland OR Syria OR Syrian OR East Timor OR East Timorese OR Tonga OR Tongan OR Turkmenistan OR Turkmen OR Tuvalu OR Tuvaluan OR Ukraine OR Ukrainian OR Uzbekistan OR Uzbek OR Vanuatu OR Vietnam OR Vietnamese OR West Bank OR Gaza OR Yemen OR Yemeni OR Yemenite OR Zambia OR Zambian OR Albania OR Argentinian OR Azerbaijan OR Azerbaijani OR Belarus OR Belarusian OR Bosnia OR Bosnian OR Botswana OR Brazil OR Brazilian OR Bulgaria OR Bulgarian OR Barbados OR Bajan OR Barbadians OR Chile OR Chilean OR China OR Chinese OR Colombia OR Columbian OR Costa Rica OR Costa Rican OR Dominica OR Dominican OR Ecuador OR Ecuadorean OR Gabon OR Gabonese OR Grenada OR Grenadian OR Iran OR Iranian OR Jamaica OR Jamaican OR Jordan OR Jordanian OR Kazakhstan OR Kazakhstani OR Latvia OR Latvian OR Lebanon OR Lebanese OR Libya OR Libyan OR Macedonia OR Macedonian OR Malaysia OR Malaysian OR Maldives OR Maldivian OR Mauritius OR Mauritian OR Mexico OR Mexican OR Montenegro OR Montenegrin OR Namibia OR Namibian OR Palau OR Palauan OR Peru OR Peruvian OR Russia OR Russian OR Serbia OR Serbian OR South Africa OR South African OR Saint Lucia OR Saint Vincent OR Suriname OR Surinamer OR Thailand OR Thai OR Tunisian OR Turkey OR Uruguay OR Venezuela OR Venezuelan).

### Type-two diabetes

(“Type-two Diabetes Mellitus” [tw] OR “Noninsulin-Dependent” [tw] OR “Diabetes Mellitus” [tw] OR “Non Insulin Dependent” [tw] OR “Non-Insulin-Dependent” [tw] OR “Non-Insulin-Dependent Diabetes Mellitus” [tw] “Diabetes Mellitus, Type II” [tw] OR “NIDDM” [tw] OR “Diabetes Mellitus, Noninsulin Dependent” [tw] OR “Diabetes Mellitus, Maturity-Onset” [tw] OR “Diabetes Mellitus, Maturity Onset” [tw] OR “Maturity-Onset Diabetes Mellitus” [tw] OR “Maturity Onset Diabetes Mellitus” [tw] OR “Diabetes Mellitus, Slow-Onset” [tw] OR “Diabetes Mellitus, Slow Onset” [tw] OR “Type 2 Diabetes Mellitus” [tw] OR “Noninsulin-Dependent Diabetes Mellitus” [tw] OR “Noninsulin Dependent Diabetes Mellitus” [tw] OR “Maturity-Onset Diabetes” [tw] OR “Diabetes, Maturity-Onset” [tw] OR “Maturity Onset Diabetes” [tw] OR “Type 2 Diabetes” [tw] OR “Diabetes, Type 2” [tw] OR “Diabetes Mellitus, Adult-Onset” [tw] OR “Adult-Onset Diabetes Mellitus” [tw] OR “Diabetes Mellitus, Adult Onset” [tw] OR “Type two Diabetes” [tw]).

### Diabetes self-management education

**“**management of” [tw] OR “diabetes self-management education” [tw] OR “diabetes self-management” [tw] OR “self-management education” [tw] OR “DSME” [tw].

### Implementation science outcomes

**“**adherence” [tw] OR “quality of delivery” [tw] OR “implementation” [tw] OR “acceptability” [tw] OR “adoption” [tw] OR “cost” [tw] OR “fidelity” [tw] OR “patient satisfaction” [tw] OR “uptake” [tw] OR “initial implementation” [tw] OR “intention to try” [tw] OR “implementation” [tw] OR “delivered as intended” [tw] OR “integrity” [tw] OR “quality of the program” [tw] OR “delivery” [tw] OR “marginal cost” [tw] OR “cost-effectiveness” [tw] OR “cost-benefit” [tw] OR “affordability” [tw] OR “total cost” [tw] OR “utilization” [tw].
